# Combining a weed traits database with a population dynamics model predicts shifts in weed communities

**DOI:** 10.1111/wre.12126

**Published:** 2014-11-12

**Authors:** J Storkey, N Holst, O Q Bøjer, F Bigongiali, G Bocci, N Colbach, Z Dorner, M M Riemens, I Sartorato, M Sønderskov, A Verschwele

**Affiliations:** * Agroecology Department, Rothamsted ResearchHarpenden, UK; † Department of Agroecology, Aarhus UniversitySlagelse, Denmark; ‡ Istituto di Scienze della Vita, Scuola Superiore Sant'Anna – Piazza Martiri della LibertàPisa, Italy; § INRA, UMR1347 AgroécologieDijon, France; ¶ Institute of Plant Protection, Faculty of Agriculture & Environmental Science, Szent Istvan UniversityGodollo, Hungary; ** Wageningen University & Research Centre, Agrosystems ResearchWageningen, the Netherlands; †† Istituto di Biologia Agroambientale & Forestale, CNRPadua, Italy; ‡‡ Federal Research Centre for Cultivated Plants, Julius Kuhn InstituteBraunschweig, Germany

**Keywords:** demographic model, weed management, functional ecology, agricultural intensification, community assembly theory

## Abstract

A functional approach to predicting shifts in weed floras in response to management or environmental change requires the combination of data on weed traits with analytical frameworks that capture the filtering effect of selection pressures on traits. A weed traits database (WTDB) was designed, populated and analysed, initially using data for 19 common European weeds, to begin to consolidate trait data in a single repository. The initial choice of traits was driven by the requirements of empirical models of weed population dynamics to identify correlations between traits and model parameters. These relationships were used to build a generic model, operating at the level of functional traits, to simulate the impact of increasing herbicide and fertiliser use on virtual weeds along gradients of seed weight and maximum height. The model generated ‘fitness contours’ (defined as population growth rates) within this trait space in different scenarios, onto which two sets of weed species, defined as common or declining in the UK, were mapped. The effect of increasing inputs on the weed flora was successfully simulated; 77% of common species were predicted to have stable or increasing populations under high fertiliser and herbicide use, in contrast with only 29% of the species that have declined. Future development of the WTDB will aim to increase the number of species covered, incorporate a wider range of traits and analyse intraspecific variability under contrasting management and environments.

## Introduction

A number of ecological databases have been compiled of the morphological and life history traits of European floras (Fitter & Peat, 1994; Kuhn *et al*., 2004; Kleyer *et al*., 2008) and used to analyse the broad differences in ecological strategies between species that determine community assembly in contrasting habitats (Grime *et al*., 1997; Liira *et al*., 2008). Several authors have recently identified the potential for extending these trait-based approaches to agricultural systems to predict the response of weed communities to changes in management (Booth & Swanton, 2002; Fried *et al*., 2009; Hawes *et al*., 2009; Smith *et al*., 2010; Storkey *et al*., 2010; Gunton *et al*., 2011; Navas, 2012; Pinke & Gunton, 2014), or the role of weeds in delivering ecosystem services (Storkey, 2006; Moonen & Barberi, 2008; Storkey *et al*., 2013). Realising this potential will depend on collating data on weed traits and developing analytical frameworks that are able to predict the filtering effects of selection pressures on the relevant response traits. In this paper, we introduce a new web-based weed traits database (WTDB) and demonstrate how the impact of management on weed communities can be predicted using relationships between traits and parameters in a demographic model. This will involve a number of logical steps:

1. Define the specific filters associated with a given management scenario, for example growing a new crop may involve different timings of cultivation and harvest, herbicide spectrum and level of crop competition.
2. Identify the plant traits that respond to these filters, where traits are defined as ‘any morphological, physiological or phenological feature that can be measured at the level of the individual’ (Violle *et al*., 2007). For example, the response of weeds to an earlier harvest date will be mediated, in part, by the date of maturity. Where the community response is a product of multiple traits, this may involve using simulation models of weed growth and population dynamics to identify the most important parameters and the traits with which they are correlated (Colbach *et al*., 2010; Gardarin *et al*., 2010a).
3. Quantify values of the relevant traits for the candidate species in a given species pool and their relationships with demographic parameters.
4. Apply methodologies that predict the filtering effect of selection pressures associated with drivers on weed traits. This may be achieved using simple demographic models or statistical models based on empirical observations of shifts in functional metrics including community weighted means or functional diversity (Diaz *et al*., 2007; Violle *et al*., 2007). For this latter approach, data are required on relative abundance of species in the community.

A recent review of the functional approach to weed management by Gaba *et al*. (2013) provides a comprehensive assessment of the weed traits that respond to different management filters (steps 1 & 2). Initially, rather than trying to capture all of these traits, when setting up the WTDB, we chose to focus on a subset of traits that could be linked directly to function via correlations with parameters in a simple weed demographic mode, with a focus on regenerative traits. Future development of the WTDB will aim to incorporate more traits from the established growth phase, for example specific leaf area. The relationships between traits and model parameters were used to build a generic model of weed population dynamics that operated at the level of functional traits. In so doing, we address steps 3 and 4, establishing an evidence base to facilitate functional analyses of European weed floras and developing an analytical framework to capture the effect of selection pressures on a weed flora.

The ecological trait databases cited above include the majority of the European weed flora, which raises the question of why a dedicated weed traits database is required. While it is true that data on some traits, such as seed weight, will be useful to weed scientists, there are three reasons why these databases are generally inadequate to predict functional shifts in weed communities. Firstly, existing databases are largely based on data from seminatural habitats. In contrast, arable weeds exist in a highly managed, disturbed environment and the selection of input fields in a weed traits database will, therefore, be strongly influenced by the crop management context, with more of an emphasis on annual species and regenerative traits. Secondly, existing databases tend to be broad and shallow, in that the intention has been to include as much of a regional or national flora as possible with data on ecological and life history traits that are widely available from the botanical literature. In contrast, a functional analysis of weed floras will require a narrow, deep database that contains more detailed ecophysiological information on the subset of a flora that are adapted to persist in the highly disturbed habitat of arable fields. Finally, many plant traits, for example duration of flowering, are plastic and a trait recorded in one habitat may not be applicable to another (Chevin *et al*., 2010; Albert *et al*., 2012). In the case of weeds, this means data may be site and crop specific and this information needs to be incorporated into the database.

This paper reports on the development (including the rationale behind the selection of the input fields) of the WTDB that meets these criteria, by a consortium of weed scientists from eight European countries and an initial analysis of the data. The WTDB was designed to include both the parameters required by the model and the weed traits that were expected to underlie these empirical functions; the distinction between parameters and functional traits is made explicit within the WTDB by identifying traits with underlined names. The potential usefulness of the combination of the database with the trait-based weed population dynamics model is then demonstrated by applying it to the case study of recent changes in an arable flora in response to two management filters associated with agricultural intensification.

## Materials and methods

### Selection of parameters and traits

**Figure 1:**
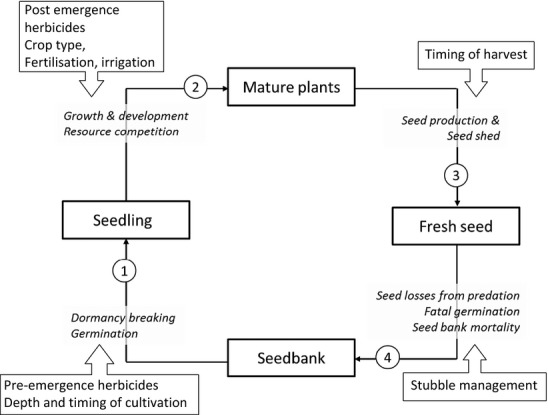
General scheme of annual weed life cycle for defining required parameters for generic demographic model indicating the processes that determine transitions between states and the point of impact of different drivers associated with changes in management. Numbers refer to transitions between stages to which the traits and parameters listed in Table 1 relate.

To identify the required fields for the WTDB, a general scheme for modelling the demographics of an annual weed was used that divided the life cycle into four states: viable seedbank, seedlings, mature plants and fresh seed. This framework could be used to specify the point in the life cycle impacted by different crop management factors and the corresponding model parameters, or related traits, that describe the transition between states and response to management (Fig.1, Table 1).

**Table 1:** Description of functional traits, and the model parameters they relate to, in the WTDB grouped according to the transitional stages in the life cycle (see Fig.1). Traits are indicated by underlined names

Trait/parameter	Description
*1. Seedbank to seedlings*	
GERMBASE	Base temperature for germination (°C).
GERMCHILL	Chilling requirement to break primary dormancy. Either ‘absolute’, ‘partial’ or ‘none’.
GERMLIGHT	Light requirement for germination. Either ‘absolute’, ‘partial’ or ‘none’.
EMCAL[1..12]	Emergence calendar; relative percentage emergence for each month (1–12) totalling 100% for whole years.
EMTOT[1,2]	Total percentage emergence per year from seedbank of known size in [1] disturbed and [2] undisturbed soil.
*2. Seedlings to mature biomass*	
COMPHEIGHT	Maximum plant height at maturity in cm.
COMPHYP[1,2]	Hyperbolic yield loss equation*: yield loss per plant at low density, *i* [1] and maximum yield loss at high weed density, *m* [2].
PHENJUV[1..4]	Duration of juvenile stage. The duration was specified by average [1] and variance [2]. Where thermal time or photothermal time was used, base temperature [3] and base day length [4] were also included.
*3. Mature biomass to fresh seeds*	
SEEDWEIGHT	Air-dried 1000 seed weight in g.
FECUNDITY[1,2]	Seed production in relation to mature plant biomass from a plot of Ln (seed production) on Ln (shoot biomass): slope [1] and intercept [2].
PHENFLO[1..4]	Duration of flowering stage.
PHENMAT[1..4]	Duration of seed maturation stage.
*4. Fresh seed to seedbank*	
SEEDCOAT	Percentage of total seed weight made up of non-investing structures (e.g. seed coat, awns, pappus).
SEEDPER[1,2]	Half-life of seedbank in [1] undisturbed and [2] disturbed soil, measured in years, assuming an exponential decay.
EMDEPTH[1,2]	Soil depth for emergence as the optimum [1], that is the depth from which maximum emergence was observed, and maximum [2], that is the deepest layer from which emergence was observed (cm).

* % yield loss = (*i* * weed density)/(1 + *i* * weed density/*m*).

The philosophy behind the WTDB was to compile data that were sufficiently comprehensive to describe the entire annual weed life cycle illustrated in Fig.1 and were also available in the literature for a wide range of species. The level of detail captured within any one process was, therefore, deliberately constrained and the selection criteria for the database input fields determined using expert opinion on the availability of data on traits and the parameters for a small number of empirical functions that are well established in the literature.

1. *Seedbank to seedlings*. The number of seedlings emerging at a given time will be a product of seed dormancy characteristics and the environmental conditions (light, temperature, nitrogen and moisture). Emergence calendars that quantify the percentage of total emergence of weeds in each month are common in the literature. These calendars were included in the database with 12 fields for each month (EMCAL[1..12]), along with data on the physiological traits that may help to determine these observed patterns: base temperature for germination (GERMBASE), chilling and light requirements (GERMCHILL, GERMLIGHT). Where there were data on the proportion of a known seedbank size emerging, these were also included, specifying whether the data were from disturbed or undisturbed soil (EMTOT[1,2]).
2. *Seedlings to mature biomass*. The final weed biomass at maturity will be determined by the competitive balance between the weed and the crop. This can be described empirically by a hyperbolic yield loss equation (Cousens, 1985), Table 1, that has been widely validated and parameterised for many weed species. The equation models percentage yield loss using two parameters, *i* (percentage yield loss per weed plant per unit area as density approaches zero) and *m* (the upper limit of yield loss), which were included in the database (COMPHYP[1,2]). These parameters will partly be crop and site specific (Lindquist *et al*., 1999), and so, as with all the entries in the WTDB, information was included on the crop type and location of the experiment from which the data were derived. The relationship between crop yield loss and weed biomass at maturity tends to be conserved within a crop, regardless of the identity of the weed species (Olsen *et al*., 2005); *i* and *m* can, therefore, be used to predict mature weed biomass (Rasmussen & Holst, 2003). A previous sensitivity analysis of a mechanistic weed/crop competition model identified weed height as an important trait determining relative competitive performance (Kropff *et al*., 1992), and this was, therefore, also included (COMPHEIGHT).
3. *Mature biomass to fresh seeds*. The allometric, linear relationship between Ln shoot biomass at maturity and Ln seed production is now well established as a way of modelling fecundity, and parameters are available in the literature for many weed species (Thompson *et al*., 1991). The slope and the intercept for each species included in the database were entered as FECUNDITY[1,2]. It was expected that the relationship between biomass and seed production would be mediated by seed weight with smaller seeded weeds generally being more fecund. To quantify this relationship, air-dried seed weight was included as an additional trait, SEEDWEIGHT.
4. *Fresh seeds to seedbank*. The proportion of the seedbank that is available to produce seedlings in the following season (*t*_*+1*_) will be determined by the rate of decline of the seedbank due to mortality which can be described using an exponential function, Equation 1

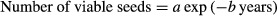
(1)

where *a* = number of seeds at time *t* after subtracting germinated seed and *b* is parameter quantifying rate of exponential decline. The half-life of the seedbank in years, defined by Ln (2)/*b*, was included as a field in the database, again specifying whether the data were from disturbed or undisturbed soil, SEEDPER[1,2]. It has recently been proposed that seed mortality may be related to the thickness of the seed coat (Gardarin *et al*., 2010b). The proportion of the seed weight made up of non-investing structures will also be relevant when relating seed size to processes mentioned above, including the depth of germination and fecundity and where data on this variable were available, they were included (SEEDCOAT). The proportion of the seedbank that is viable will also be related to the stratification of seeds by cultivation and the regenerative niche defined by the available soil horizon for successful emergence. The depth of optimum emergence and the maximum depth of emergence (EMDEPTH[1,2]) were, therefore, also included in the database.

Finally, three sets of parameters were introduced to summarise the duration of the developmental stages: juvenile (PHENJUV[1..4]), flowering (PHENFLO[1..4]) and seed maturation on the plant (PHENMAT[1..4]). The duration was specified by average [1] and variance [2]. The timescale could be days, day-degrees or photothermal time, in which daily day-degrees are multiplied by (day length minus base day length). As needed, the base temperature for day-degree calculation was given [3], together with base day length [4].

### Database design and data entry

The WTDB has been implemented as a relational database on Microsoft SQL Server 2000 (+2008). The web interface was developed in Microsoft ASP.NET 2.0 (C no.) (Microsoft corporation, Redmond, WA, USA) and is accessible to anyone for viewing data, statistics and reports. Researchers who wish to add weed species and/or parameters to the database need to register to add data online; new data are screened before becoming available to the wider community. WTDB is available from the WeedML website (http://www.ipmdss.com/wtdb).

The authors intend that the WTDB becomes established as an enduring resource for the weed science community that captures data on the variables described above for as many weed species as possible. However, to validate the approach, a set of common European species were chosen to initially populate the database; at the time of writing, sufficient data have been collated to carry out an analysis for 19 species, *Abutilon theophrasti* Medick., *Alopecurus myosuroides* Huds., *Ambrosia artemisiifolia* L., *Apera spica-venti* (L.) P. Beauv., *Anisantha sterilis* Nevski., *Centaurea cyanus* L., *Chenopodium album* L., *Echinochloa crus-galli* (L.) P. Beauv., *Fallopia convolvulus* (L.) Å. Löve., *Galium aparine* L., *Papaver rhoeas* L., *Poa annua* L., *Polygonum aviculare* L., *Persicaria lapathifolia* (L.) Gray, *Raphanus raphanistrum* L., *Sinapis arvensis* L., *Solanum nigrum* L., *Stellaria media* (L.) Vill. and *Tripleurospermum inodorum* (K.Koch) Sch. Bip. For each species, a literature search was carried out and data supplemented from unpublished sources available within the consortium. Each data entry was classified in terms of the quality of the source: peer reviewed journal, conference paper, report, unpublished data or expert opinion. Where information was given in the original source on providence of the seed, crop type and location of experiments, this was also included, along with brief notes on the methodology used. If the results of a number of trials were reported in a paper, all of these data were included to capture information on the variance in a given parameter or trait.

One of the objectives of the WTDB was to quantify relationships between weed traits and plant function (such as the pattern of emergence and competitive performance) as described by model parameters. If these relationships can be established, the contrasting response of weeds with divergent trait values to perturbations in the cropping system can be predicted on the basis of trait data alone. A correlation matrix of variables in the database was therefore constructed, using mean values for items for which there were multiple values. Where data were available on the parameters from the hyperbolic yield loss equation, COMPHYP[1,2], these were used to calculate the number of individual weeds required to incur a 5% yield loss (*D*_5%_) and this value was also used in the correlation analysis.

### Proof of concept

To illustrate how the WTDB, combined with a generic weed population dynamics model, can be applied to the study of weed community assembly, we used the case study of recent changes in the arable flora in the UK in response to the two filters of increased herbicide and fertiliser use, following the four steps described in the introduction. In so doing, our intention was not to complete a comprehensive analysis of all the complex interactions of the drivers operating on the system, but to demonstrate how traits can be used to infer broad shifts in the functional make up of weed floras. Firstly, the relevant management filters and functional response traits were identified (steps 1 and 2). A consistent response of arable floras to increased fertiliser and herbicide inputs has been observed at a European scale (Storkey *et al*., 2012), and it is likely that increased agrochemical inputs are the primary driver of species declines in the UK. Analyses of the contrasting traits of rare or declining arable weeds and species that have remained common identified maximum height and seed weight as important traits that determined the response of a species to intensification (Storkey *et al*., 2010; Pinke & Gunton, 2014).

Steps 3 and 4 were completed by constructing a series of empirical equations to model the life cycle of a generic annual weed of a given height and seed weight using the correlations between these traits and the model parameters identified from the WTDB. Two parameters required by the model, the proportion of the seedbank emerging and half-life of the seedbank, were not related to any functional traits [although wider ecological theory would predict a relationship with seed weight (Thompson *et al*., 1993; Turnbull *et al*., 2004)]. As the frequency distribution of both parameters was highly skewed, with a greater proportion of low values for emergence and short seed persistence, the median value was used in the model for each variable. An alternative approach would be to use stochastic algorithms and perform multiple runs that randomly sample values from the fitted frequency distribution for each parameter.

The life cycle model was initialised with 1000 seeds m^−2^ of which 71 m^−2^ were predicted to emerge on the basis of the median value for EMTOT from disturbed soils in the WTDB. A proportion of these seedlings were killed by herbicide (see below). The mature biomass of the surviving weeds was modelled in two steps. Firstly, the relationship between competitive ability (defined as *D*_5%_) and COMPHEIGHT was derived from the WTDB. The one parameter version of the hyperbolic yield loss equation was solved for *i* and used to predict % yield loss from a weed with a given height (Eqns 2 and 3). Secondly, mature weed biomass was calculated from a generic relationship with crop yield loss derived from unpublished data from weed competition trials at three sites and in 2 years using natural weed populations with different species identities (Figure S1).


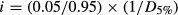
(2)


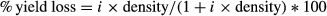
(3)

Fresh seed production was calculated from the allometric relationship between Ln (mature biomass) and Ln (seed production) assuming a regression line of 1. The intercept of the regression (seeds produced by a plant of 1 g) was calculated from the relationship with SEEDWEIGHT in the WTDB. A proportion of the fresh seed will be lost to predation and fatal germination. The fitness of the population (*λ*) of a virtual weed species, over a single growing season, with a given maximum height and seed weight was calculated as seed number m^−2^ at time *t*_*+*1_ divided by seed number at time *t*, after losses in the seedbank had been accounted for using a median value for seedbank half-life (1 year) from the WTDB. The parameter value for seed losses in the model (79%) was chosen in combination with the percentage of seedlings killed by herbicide in the high use scenario (96%), see below, to simulate the maximum feasible stress on the system. This was defined as the scenario in which the shortest model plant in the system (10 cm) was able to maintain a viable population by reducing seed size and increasing fecundity under conditions of high herbicide mortality and fertility.

The life cycle model was used to predict the fitness of generic weeds with different combinations of maximum height and seed weight under four scenarios with either high (0.96) or low (0.5) herbicide mortality and fertiliser use. The effect of fertiliser was modelled using a function describing the relationship between relative competitive ability at high and low fertility, weed seed weight and height relative to the crop (using a crop height of 80 cm) parameterised in a previous study (Storkey *et al*., 2010). Competitive ability at low fertility was measured in soil with a range of 0–60 kg N ha compared with the high fertility treatments which had a range of 120–480 kg N ha. This coefficient was used to adjust the percentage crop yield loss in the model. For each scenario, height was incremented in 10 cm intervals between 10 and 200 cm and seed weight in 0.01 mg intervals between 0.01 and 20 mg and *λ* calculated for all combinations. The output of the model was plotted as fitness contours. The responses of populations of individual weed species were then mapped onto the contour plots, using values for height and seed weight derived from the Ecoflora Database for height (Fitter & Peat, 1994) and the Seed database held at Kew Gardens, UK, for seed weight (Flynn *et al*., 2004).

Two sets of weed species were projected onto the fitness contours using only data for height and seed size. Firstly, a list of common species was derived from a survey of the weed flora in 65 fields of winter oilseed rape in the UK as part of the Farm Scale Evaluations (FSE) of genetically modified (GM) crops (Bohan *et al*., 2005). Because the counts in the GM crop treatment were recorded in September and October before the application of herbicide, they represent a useful nationwide survey taken over 3 years of weed communities that are currently typical of autumn-sown crops in the UK. Secondly, a list of weeds that are currently rare or threatened and reflect a large negative population change index between 1960 and 2000 (Preston *et al*., 2002) was taken from a recent report of the threat status of Britain's vascular plants (Cheffings & Farrell, 2005). In both cases, obligate spring-germinating species were excluded. Species in the WTDB were excluded to ensure a completely independent data set from the one used to derive the fitness contours. The full list of species with trait values and calculated *λ* under the present day scenario of high inputs are presented in Supporting Information Table S1.

## Results

Over 1500 data values were entered into the WTDB for 19 weed species. Coverage varied between the species and database fields. Complete data sets were compiled for well-studied species, such as *G. aparine* and *A. myosuroides,* with several data entries from a number of separate data sources available for most database fields. However, it was not possible to find data on some variables for species that are reported on less commonly in the literature. In particular, values for the parameters describing competitive ability, seed persistence and phenology were often not available (Table 2). Where several values were obtained for a single database field, it was possible to quantify variance within parameters or traits in terms of the maximum value as a percentage of the minimum. Values for yield loss per plant per unit area and percentage germination appear to be particularly variable on this basis for a given species, whereas the parameters describing the relationship between biomass and fecundity and values for seed weight are more stable between data sources.

**Table 2:** Summary of data entered into WTDB for 19 species for which data sets are most complete. Minimum and maximum values are presented for each field with number of data entries in parenthesis

Species	Maximum height (cm)	% Yield loss/Plant m^−2^	Fecundity*	Germination base temperature (°C)	Max. depth of emergence (cm)	Percentage emergence†	Seed persistence‡	Seed weight (mg)
*Abutilon theophrasti*	97–300 (10)	0.9–60.3 (23)	2.48–3.31 (2)	6.2–6.5 (2)	12 (1)	1.5–1.9 (3)	0.79 (1)	9.0–10.6 (4)
*Alopecurus myosuroides*	115 (1)	0.02–1.43 (17)	4.93 (1)	0.0 (1)	8–12 (2)	40.4 (1)	0.34–1.85 (6)	1.55–2.19 (6)
*Ambrosia artemisiifolia*	120–180 (3)	7.07–25.9 (7)	–	5.0–6.0 (2)	4–6.5 (3)	–	–	1.72–3.99 (10)
*Apera spica-venti*	100 (1)	0.08–3.4 (7)	6.55 (1)	–	1–3.5 (2)	30 (1)	0.43 (1)	0.1–0.1 (2)
*Anisantha sterilis*	70–100 (5)	–	4.51 (1)	3.0 (1)	4.5–13 (4)	65 (1)	1.0 (1)	5.26–8.37 (7)
*Centaurea cyanus*	60–80 (7)	0.11–0.25 (3)	–	1.7–5.0 (4)	3–10 (7)	11–20 (3)	5.0 (1)	3.28–4.80 (7)
*Chenopodium album*	60 (1)	–	6.04–7.36 (4)	5.8 (1)	6 (1)	7.8–15.8 (2)	–	0.49–0.79 (6)
*Echinochloa crus-galli*	75–110 (2)	–	6.15 (1)	6.2–13 (5)	7.5 (1)	0–100 (4)	0.69 (1)	0.89–2.35 (6)
*Fallopia convolvulus*	64 (1)	–	4.55 (1)	–	9.5–19 (2)	–	–	5.06–7.0 (4)
*Galium aparine*	67–180 (3)	0.09–23 (44)	3.12–5.09 (3)	0.0 (1)	10 (1)	58 (1)	0.76–1.90 (3)	6.64–13.06 (5)
*Papaver rhoeas*	88 (1)	0.1 (1)	6.38–7.03 (2)	1.0 (1)	–	3–70 (2)	5–44 (2)	0.07–0.20 (4)
*Poa annua*	38 (1)	–	–	–	–	35 (1)	–	0.19–0.48 (11)
*Polygonum aviculare*	58 (1)	–	4.66 (1)	0.0–8.0 (2)	3–16 (2)	32 (1)	–	0.68–3.0 (7)
*Persicaria lapathifolia*	–	–	–	1.7 (1)	6.5 (1)	50.1 (1)	–	1.50–2.91 (6)
*Raphanus raphanistrum*	200 (1)	0.51–1.71 (6)	–	5 (1)	–	1.4–10.4 (4)	1.0–1.0 (2)	2.10–4.76 (3)
*Sinapis arvensis*	93–99.5 (3)	0.3–7.0 (4)	3.6–4.93 (3)	10 (1)	–	20–80 (4)	2.3 (1)	0.92–2.33 (7)
*Solanum nigrum*	24.9–78.1 (4)	–	7.34 (1)	7.5–11.5 (4)	6–15 (2)	–	0.86 (1)	0.70–1.02 (8)
*Stellaria media*	61.5 (1)	0.016–4.2 (32)	5.2–6.46 (4)	1.4–4.7 (2)	5–10.5 (4)	1.7–30 (3)	1.46–1.7 (2)	0.33–0.67 (9)
*Tripleurospermum inodorum*	100–109 (2)	1.31 (1)	6.31–6.67 (3)	1.9 (1)	8 (1)	–	1.6 (1)	0.27–0.74 (15)

* *y-intercept* of allometric linear relationship between Ln (seed production) with Ln (shoot biomass at maturity).

† Total germination per year in disturbed soil expressed as percentage of seedbank.

‡ Half-life of seedbank in disturbed soil measured in years assuming an exponential decay.

**Figure 2:**
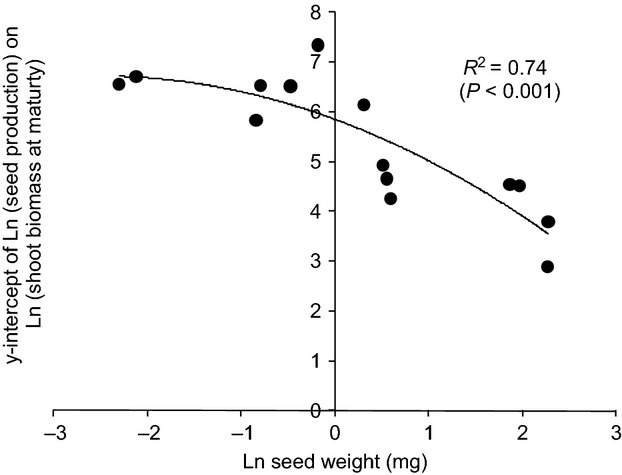
Relationship between Ln seed weight (mg) and *y-*intercept of allometric relationship between Ln (seed production) and Ln (biomass at maturity), equivalent to Ln numbers of seeds produced by 1 g biomass. A second order polynomial has been fitted to the data, *y* = −0.14*x*^2^ − 0.70*x* + 5.85. Where multiple values were available for a given species, means were used.

A number of significant correlations were found between functional traits and model parameters. The slope of the allometric relationship between the natural logarithm of seed production and shoot biomass at maturity was close to one for most species, indicating that size dependency of reproductive allocation remains constant (Sugiyama & Bazzaz, 1998). However, there were large interspecific differences in the *y-*intercept of the function, and these were strongly correlated with seed weight (Fig.2). A quadratic function gave a better fit to the data than a straight line, indicating that at very small seed sizes, additional physiological constraints may be limiting further increases in fecundity. There was also a significant relationship with maximum height, but with less variance explained (30%).

**Figure 3:**
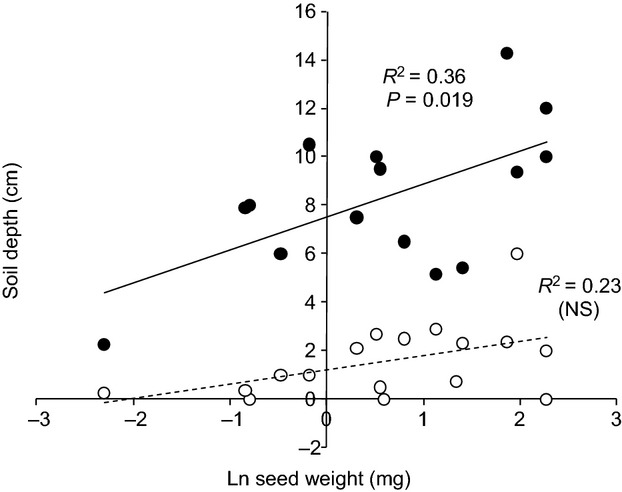
Relationship between Ln seed weight (mg) and depth of successful germination. • Maximum depth, *y* = 1.36*x* + 7.53. ○ Optimum depth (NS relationship). Where multiple values were available for a given species, means were used.

**Figure 4:**
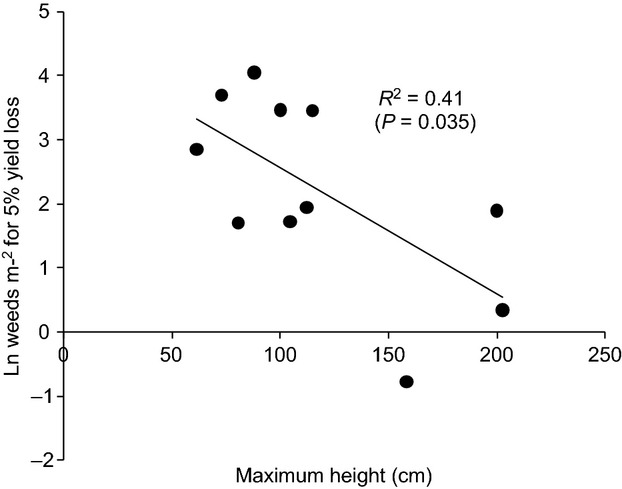
Relationship between maximum height and number of weed plants required to incur a 5% yield loss using mean values for the parameters in the Cousens equation, YL = (*i* D)/(1 + *i* D/*m*) – log transformed to make relationship linear; *y* = 0.020*x* + 4.51.

Seed weight was also significantly correlated with maximum depth of emergence (Fig.3), indicating that the soil layer available for successful emergence is greater for species with larger seeds. The data for the optimum depth of germination were much less variable between species, generally being <3 cm, and the correlation with seed weight was not significant (Fig.3). There was a significant correlation between maximum height and the weed density that results in a 5% yield loss (expressing the data in this way combined the effect of the two parameters in the hyperbolic yield loss equation; Fig.4). However, the data entries for height, *i* and *m* were both variable within a species (Table 2). Finally, analysis of the germination calendar data suggested that it was possible to identify two groups of species (obligate spring germinators vs. generalists) and that these could be separated on the basis of the base temperature for germination and chilling requirement. (Figure S1). However, detail on dormancy characteristics is currently lacking in the database, and there is potential to integrate recent studies that relate dormancy to traits such as seed coat thickness into our approach (Gardarin *et al*., 2012).

**Figure 5:**
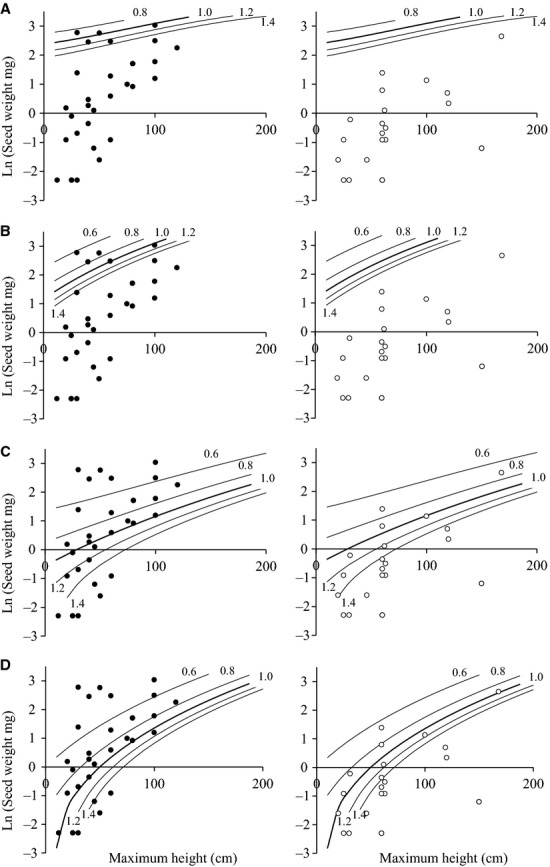
Output of the life cycle model for a generic annual weed, with different combinations of seed weight and maximum height, expressed as fitness contours indicating population growth (*λ* > 1) or decline (*λ* < 1) for four different scenarios: (A) low herbicide mortality, low fertility, (B) low herbicide mortality, high fertility, (C) high herbicide mortality, low fertility and (D) high herbicide mortality, high fertility. Data on the height and seed weight of two sets of weed species have been mapped onto the contour plots: • rare or declining arable weed species and ○ species commonly found in UK winter crops.

A sensitivity analysis of the generic life cycle model (quantifying the effect of incrementing each parameter by 5% on *λ*) indicated that changing the intercept of the relationship between Ln (mature biomass) and Ln (seed number) had a disproportionate effect, resulting in a change of 19.6% in *λ*. Using the relationships derived from the WTDB to populate a series of empirical equations describing the weed life cycle, successfully captured a proportion of the filtering effect of increased intensification on the UK arable flora (Fig.5). In the scenario with low herbicide and fertility, all but two of the weed species were predicted to have a *λ* > 1. Increasing herbicide pressure in particular selected against the majority of the rare or declining species. In the present day scenario with high inputs, 17 of the 22 common species (77%) were predicted to have *λ* ≥ 1. This contrasts with the 31 rare or declining species, of which only 9 (29%) were predicted to have *λ* ≥ 1 (Supporting Information Table S1).

## Discussion

Weed population dynamics models have been criticised in the past for promising much in terms of predicting absolute numbers of weeds under different scenarios, but delivering little in terms of science that is of practical benefit (Holst *et al*., 2007; Moss, 2008). The development of the WTDB, combined with weed demographic models, attempts to address this concern. We acknowledge that predicting the fine scale dynamics of a single species in a field with sufficient accuracy to inform control decisions without prohibitively detailed measurements of environmental variables remains a significant challenge (Freckleton *et al*., 2008). What we are proposing, however, is a broader approach using data on the functional traits that respond to management filters and, where appropriate, modelling, to predict *qualitative* shifts in the flora. That is, for a given change in management, it should be possible to identify groups of species that are either negatively or positively selected for by the associated drivers based on data on the relevant traits (Booth & Swanton, 2002).

The correlations between traits and model parameters in the WTDB support this concept of using a functional approach to predicting the filtering effect of management changes on weed communities. Although the simple model was useful for testing the hypothesis that increased inputs have selected between species on the basis of seed weight and maximum height, there remained a significant number of false positives and negatives. Declining species that were predicted to have sustainable populations (‘false positives’) occupied the small seed, short stature trait space and tended to be stress tolerant, calcifuge ruderals, including *Filago lutescens* and *Arnoseris minima*, that are characterised by late flowering. It is likely that the level of seed production predicted by the model may, therefore, not be achieved before crop harvest. There were also a number of ‘false negatives’ (common species that were predicted to have declining populations). In part, this may be explained by intraspecific variability in traits, particularly height. However, it is also likely that other factors may be responsible, for example decreased sensitivity to herbicides or a greater competitive ability than predicted by the model.

In our example, the modelling of the selection pressures of herbicides and fertilisers has been kept deliberately simple and does not capture many processes, for example differential susceptibility of species to herbicides (which would need to be modelled at the species level). In addition, the interaction of some of filters on the functioning of the system, for example the relative competitive ability of weeds, may require some processes to be modelled mechanistically. Integrating the functional approach with process-based models would also be an important step in predicting the interaction of environmental drivers with management, for example under climate change (Stratonovitch *et al*., 2012). The lack of detailed data in the literature on weed phenology to parameterise PHENFLO and PHENMAT is also currently a constraint and is a knowledge gap that needs addressing to predict the ability of weeds to complete their life cycle in different scenarios and their capacity to adapt (see below). A simpler approach using data on flowering times and flowering duration available in national floras may partly address this gap. The model presented here only increments two traits and is deterministic; a future research aim is to generate fitness contours in multidimensional space using more traits and to incorporate uncertainty in model parameters, such as seed losses to predation, using stochastic approaches.

Our approach has two major assumptions. Firstly, for a management driver to select between species, intraspecific variability must be smaller than interspecific variability. There were not sufficient data within the WTDB to formally test this assumption but remains a future objective as the database expands. One reason for the intraspecific variability that is present in data entries in the WTDB is that data were derived from a wide range of crops and geographic regions. It is possible to filter the data according to crop and region and, as the amount of data contained within the WTDB increases, it will increasingly be possible to generate data sets that are applicable to specific cropping system. The second, and related, assumption is that the rate of decline of a species under negative selection pressure is greater than their capacity to adapt (Neve *et al*., 2009; Clements & DiTommaso, 2011), meaning a species can effectively move within the fitness space. We suggest that the approach of generating plots of fitness contours based on the response of generic weeds with contrasting trait values and mapping species onto the resulting ‘fitness space’ has the flexibility to address both the issue of intraspecific variability and adaptation. If enough data are available on variability between populations of a species in trait values, as opposed to mapping the species onto the predicted fitness space as a point, gradients or frequency distributions of trait values could be used. This would indicate the potential for individual populations of a given species to persist under negative selection pressure, as well as the potential direction of future adaptation.

## Conclusion

Our intention has been to make the WTDB as complete as possible for the traits and species currently included. However, additional progress is required before its full potential as a tool for understanding weed community dynamics can be fully realised. Firstly, it is likely that the identity and number of input fields may need to be refined, to balance the need for comprehensive data for a wide range of species with the requirements of models that simulate the impact of multiple management drivers at varying levels of complexity. For example, leaf traits determine the response of species to the frequency of soil disturbance would be a valuable addition to the database (Westoby, 1998; Gaba *et al*., 2013). Secondly, this paper has reported on the initial design and filling of the database for the first 19 species (currently restricted to annuals) as proof of concept. However, the scope of the database could be expanded to include many more weed species and to fill in gaps where data are missing. This will largely rely on the WTDB being dynamic, in that new data should continually be entered and analysed by the international weed science community as new experiments are carried out. Finally, it would be valuable to further validate the functional approach against additional examples from the large published literature on weed community shifts under changing management, including changes in the relative abundance of common species (McCloskey *et al*., 1996; Barberi *et al*., 1997; Hyvonen & Salonen, 2002; Legere *et al*., 2005; Smith & Gross, 2007; Andreasen & Stryhn, 2008). This will involve the integration of multiple drivers of weed community composition and capture more subtle effects acting on shorter timescales. Such an exercise would also inform future modifications and additions to the WTDB.

## Supporting Information

**Figure S1** Germination calendars for weed species grouped as either a) obligate spring germinating or b) generalists with base temperatures for germination in parenthesis. Only data from the northern hemisphere have been included. **Polygonum persicaria* has an absolute chilling requirement for germination. **Figure S2** Relationship between weed biomass and crop yield loss in autumn drilled crops in the UK. Data were derived from weed competition trials done at three sites and 2 years (2002–3 and 2003–4) using natural weed populations. The sites were: Boxworth (52.25N, 0.032W), Terrington (52.77N, 0.318W) and High Mowthorpe (54,11N, 0.644W) and covered a range of soil types. Each trial consisted of full control plots, untreated plots and ‘managed plots’ in a fully randomised design with ten replicate blocks, plot size 3 × 12 m. The full control plots were treated with a conventional herbicide programme for the weed species present and the untreated plots received no herbicide, except where aggressive grass-weeds were present, for which an appropriate graminicide was applied. Decisions whether to apply herbicides to the ‘managed’ plots were based on data from autumn plant counts. If the weeds were expected to incur >5% yield loss, the plots were sprayed. Data are presented on the mean weed biomass in June and crop yield loss with error bars indicating the standard errors from ten replicates at each site. Despite the trials having different weed communities and being done at different sites and years, a single regression line could be fitted to the relationship. **Table S1** List of common and rare/declining species mapped onto fitness contour plots with values used for maximum height, seed weight and predicted *λ* for the high fertiliser, high herbicide scenario (only species not included in the analysis of WTDB were used for the validation exercise).
