# Supplementary material for: Combining a weed traits database with a population dynamics model predicts shifts in weed communities
Source: Weed Res. 2014 Nov 12;55(2):206–18. doi: 10.1111/wre.12126 (PMC4480327; doi:10.1111/wre.12126)
Supplement: Supplementary file 1 [file wre0055-0206-sd1.doc]

**Supporting Information**

**Fig. S1** Germination calendars for weed species grouped as either a) obligate spring germinating or b) generalists with base temperatures for germination in parenthesis. Only data from the northern hemisphere have been included. **Polygonum persicaria* has an absolute chilling requirement for germination.

a)

b)

**Fig. S2** Relationship between weed biomass and crop yield loss in autumn drilled crops in the UK. Data were derived from weed competition trials done at three sites and two years (2002-3 and 2003-4) using natural weed populations. The sites were: Boxworth (52.25N, 0.032W), Terrington (52.77N, 0.318W) and High Mowthorpe (54,11N, 0.644W) and covered a range of soil types. Each trial consisted of full control plots, untreated plots and ‘managed plots’ in a fully randomised design with ten replicate blocks, plot size 3 x 12m. The full control plots were treated with a conventional herbicide programme for the weed species present and the untreated plots received no herbicide, except where aggressive grass-weeds were present, for which an appropriate graminicide was applied. Decisions whether to apply herbicides to the ‘managed’ plots were based on data from autumn plant counts. If the weeds were expected to incur greater than 5% yield loss, the plots were sprayed. Data are presented on the mean weed biomass in June and crop yield loss with error bars indicating the standard errors from ten replicates at each site. Despite the trials having different weed communities and being done at different sites and years, a single regression line could be fitted to the relationship.

**Table S1** List of common and rare / declining species mapped onto fitness contour plots with values used for maximum height, seed weight and predicted λ for the high fertiliser, high herbicide scenario (only species not included in the analysis of WTDB were used for the validation exercise)

| **Common species** | **Maximum height (cm)** | **Seed weight (mg)** | **λ** |
| --- | --- | --- | --- |
| *Aethusa cynapium* | 120 | 1.4 | 2.83 |
| *Aphanes arvensis* | 20 | 0.2 | 1.04 |
| *Avena fatua* | 168 | 14.1 | 0.97 |
| *Capsella bursa-pastoris* | 60 | 0.1 | 2.35 |
| *Fumaria officinalis* | 100 | 3.1 | 1.15 |
| *Geranium dissectum* | 60 | 2.2 | 0.82 |
| *Geranium molle* | 62 | 1.1 | 1.15 |
| *Lamium purpureum* | 31 | 0.8 | 0.85 |
| *Lolium multiflorum* | 119 | 2 | 2.15 |
| *Matricaria discoidea* | 30 | 0.1 | 1.34 |
| *Matricaria recutita* | 60 | 0.1 | 2.35 |
| *Myosotis arvensis* | 63 | 0.4 | 1.80 |
| *Senecio vulgaris* | 46 | 0.2 | 1.61 |
| *Solanum nigrum* | 60 | 0.7 | 1.36 |
| *Sonchus asper* | 150 | 0.3 | 12.85 |
| *Sonchus oleraceus* | 150 | 0.3 | 12.85 |
| *Trifolium dubium* | 25 | 0.4 | 0.96 |
| *Urtica urens* | 60 | 0.5 | 1.57 |
| *Veronica arvensis* | 25 | 0.1 | 1.23 |
| *Veronica hederifolia* | 60 | 4 | 0.65 |
| *Veronica persica* | 63 | 0.6 | 1.53 |
| *Viola arvensis* | 60 | 0.4 | 1.71 |
| **Rare or declining species** |  |  |  |
| *Adonis annua* | 40 | 11.6 | 0.49 |
| *Agrostemma githago* | 100 | 12.1 | 0.59 |
| *Ajuga chamaepitys* | 20 | 1.2 | 0.67 |
| *Arnoseris minima* | 30 | 0.5 | 0.97 |
| *Bromus interruptus* | 100 | 3.3 | 1.10 |
| *Bromus secalinus* | 120 | 9.5 | 0.75 |
| *Bupleurum rotundifolium* | 75 | 2.7 | 0.89 |
| *Caucalis platycarpos* | 30 | 16 | 0.48 |
| *Chrysanthemum segetum* | 60 | 1.8 | 0.89 |
| *Euphorbia platyphyllos* | 80 | 2.5 | 0.98 |
| *Filago gallica* | 25 | 0.1 | 1.23 |
| *Filago lutescens* | 25 | 0.1 | 1.23 |
| *Filago pyramidata* | 30 | 0.1 | 1.34 |
| *Fumaria occidentalis* | 100 | 5.9 | 0.79 |
| *Galium tricornutum* | 80 | 5.5 | 0.68 |
| *Hypochoeris glabra* | 40 | 0.7 | 1.00 |
| *Iberis amara* | 30 | 4 | 0.55 |
| *Lathyrus aphaca* | 100 | 20.7 | 0.52 |
| *Lithospermum arvense* | 80 | 5.5 | 0.68 |
| *Myosurus minima* | 12 | 0.1 | 1.01 |
| *Papaver argemone* | 50 | 0.2 | 1.73 |
| *Ranunculus arvensis* | 60 | 11.9 | 0.51 |
| *Scandix pecten-veneris* | 50 | 15.8 | 0.49 |
| *Silene gallica* | 45 | 0.3 | 1.43 |
| *Silene noctiflora* | 45 | 1.1 | 0.90 |
| *Spergula arvensis* | 60 | 0.4 | 1.71 |
| *Thlaspi perfoliatum* | 25 | 0.9 | 0.76 |
| *Torilis arvensis* | 40 | 1.6 | 0.74 |
| *Valerianella rimosa* | 40 | 1.3 | 0.80 |
| *Veronica triphyllos* | 20 | 0.4 | 0.90 |
| *Vicia parviflora* | 60 | 3.6 | 0.68 |
|  |  |  |  |
|  |  |  |  |
|  |  |  |  |
|  |  |  |  |
|  |  |  |  |
|  |  |  |  |
|  |  |  |  |
